# Structure–function analysis defines the minimal functional C-terminal domain of the variant surface glycoprotein of *Trypanosomabrucei*

**DOI:** 10.1016/j.jbc.2025.110260

**Published:** 2025-05-22

**Authors:** Nicola G. Jones, Markus Engstler

**Affiliations:** Department of Cell and Developmental Biology, University of Würzburg, Würzburg, Germany

**Keywords:** cell surface, variant surface glycoprotein, structure–function, mutagenesis, protein domain, glycosylphosphatidylinositol (GPI) anchor, *Trypanosoma brucei*, *Trypanosoma congolense*

## Abstract

In their mammalian hosts, African trypanosomes abundantly express GPI-anchored variant surface glycoproteins (VSGs) on their cell surfaces. These provide a protective surface coat that has been studied best in *Trypanosoma brucei*. The genome of this single-celled parasite contains more than 2000 VSG genes and pseudogenes, a rich foundation based on which only one functional VSG is expressed at any given time. This allows coat exchange by antigenic variation which is an elegant means of repeatedly evading the immune response of the mammalian host. All proteins of the VSG family are composed of a larger, elongated N-terminal domain that is most exposed and a smaller C-terminal domain that is sandwiched between the N-terminal domain and the GPI-anchor, which connects the protein to the outer leaflet of the plasma membrane. While sequence variability in the N-terminal domain of different members of the VSG family is essential for antigenic variation, the role of the C-terminal domain remains less clear. Additionally, other species, such as *Trypanosoma congolense* and *Trypanosoma vivax*, do not possess a similarly structured C-terminal domain in their VSGs. Here, we systematically mutated the C-terminal domain of selected *T*. *brucei* VSGs and defined a minimal domain required for VSG function. We show that the size of the minimal C-terminal domain resembles that of *T*. *congolense* VSGs, and structured regions are not essential. We further propose that the evolutionary pressure to conserve the build of the C-terminal domain is related to functions beyond protein structure.

The bloodstream form of *Trypanosoma brucei* is covered by a homogenous, dense glycoprotein layer, which, at a given time, consists almost entirely of a single isoform of the large family of GPI-anchored variant surface glycoproteins (VSGs) (1, 2, 3). This exclusive, monoallelic, expression of a single member of the VSG family is the prerequisite for antigenic variation, whereby a regular switching of the expressed VSG changes the antigenic properties of the exposed surface coat (reviewed in: (4, 5)). The VSG repertoire available is further increased by the continuous generation of mosaic VSGs (6, 7). This allows the parasite to persist by repeatedly sidestepping the host’s immune attack.

The dense VSG coat is thought to function by shielding underlying invariant surface glycoproteins and other plasma membrane proteins from immune detection (8). The shield undergoes dynamic turnover, facilitated by the fast rates of endo- and exocytosis in this organism (9). The dynamic nature of the surface coat is especially important for the partitioning of antibody–VSG complexes through hydrodynamic flow forces to the posterior end of the trypanosome cell (10). This is the site where the flagellar pocket, the sole region of endo- and exocytosis in *T*. *brucei*, is located. Here, the antibody–VSG complexes are taken up, and the bound antibodies are degraded intracellularly, which at low antibody titers counteract the host’s immune response. An intact surface coat is not only essential when the host is present, but also under cell culture conditions, where loss of VSG expression results in cells undergoing a post-mitosis/pre-cytokinesis cell cycle arrest (11, 12).

Although much progress has been made in understanding the mechanisms controlling VSG expression and switching (13), extensive knowledge of the three-dimensional structures of VSGs initially lagged behind, in part because early structural analyses on two surface proteins (14) led to a long-held belief that *T*. *brucei* VSGs, despite low primary structure similarity, all adopt very similar structures. Findings in recent years, however, have uncovered more structural diversity and introduced the concept of structural dynamicity in the *T*. *brucei* VSG family (15, 16, 17, 18). The experimental observations are supported by AlphaFold (19) predictions of a large number of VSG structures (17).

All known VSGs consist of two protein modules, a larger N-terminal domain and a smaller C-terminal domain, with the latter harboring the GPI-anchor attachment site (20). Bioinformatic approaches have divided the large family of VSGs into different groups based on characteristics of the N- and C-terminal domains (20, 21, 22). Further refinement of the classification of N-terminal domains has been achieved more recently, based on structural features of a vast range of members of the VSG family (17). These most recent classifications have the N-terminal domains divided into two main groups, A and B, which have subsequently been divided further, respectively, into A1 and A2, with A1 containing further subgroups A1a, A1b, and A1c, and B1 and B2 (17). C-terminal domains have been subdivided into six groups (numbered 1–6) (23). The elongated N-terminal domain, which lies at the interface with the host, supports formation of the shielding layer, and its antigenic variation is essential for survival of the parasite within a host (6, 24). Initial studies on VSG structures suggested that the N-terminal domains always mediate the formation of homodimers (14, 25). Recent studies, however, show this to hold true for VSGs with an A-type N-terminal domain (16, 17, 18), whereas B-type N-terminal domains, dependent on protein concentration, appear to present as monomers or trimers in solution or when crystallized (15, 17, 26).

The C-terminal domain can contain one (type 2, 4, 5) or two (type 1, 3, 6) structured regions containing four conserved cysteines each and displaying a conserved fold (18, 20, 23, 27, 28). Structural analyses combined with diffusion measurements of VSGs with either a type 1 (two structured domains) or a type 2 (one structured domain) C-terminus have suggested that this domain confers flexibility to the protein dimer, as two main conformations of the C-terminal domain were observed in both cases (18). This flexibility in the VSG C-terminal domain might assist molecular packing of the surface coat, enabling coat integrity to be maintained despite fluctuations in VSG number that may occur during, for instance, cell division or antigenic variation (18). Interestingly, other VSG-expressing African trypanosomes, such as *Trypanosoma congolense* and *Trypanosoma vivax*, do not possess the above-mentioned conserved cysteine residues in their C-terminal domains, which are presumed to be unstructured (29, 30). VSGs of these species are therefore often referred to as lacking a C-terminal domain. The overall contribution of this domain to coat assembly and function is thus generally unclear in trypanosomes.

To test which parts of the C-terminal domain of *T*. *brucei* VSGs are essential or, in other words, to define the composition of a minimal VSG C-terminal domain, we conducted a systematic mutagenesis study. We found that the structured regions are not required for efficient expression of the VSGs. A minimal C-terminal domain consisting of a linker region and a flexible or structured GPI-anchoring region is sufficient for maintaining cell growth and thus must allow coat formation. We further propose that the evolutionary pressure to conserve the build of the C-terminal domain is related to functions beyond protein structure.

## Results

### The structured regions of *T*. *brucei* VSG C-terminal domains (CTDs) are not required for coat formation

**Figure 1:**
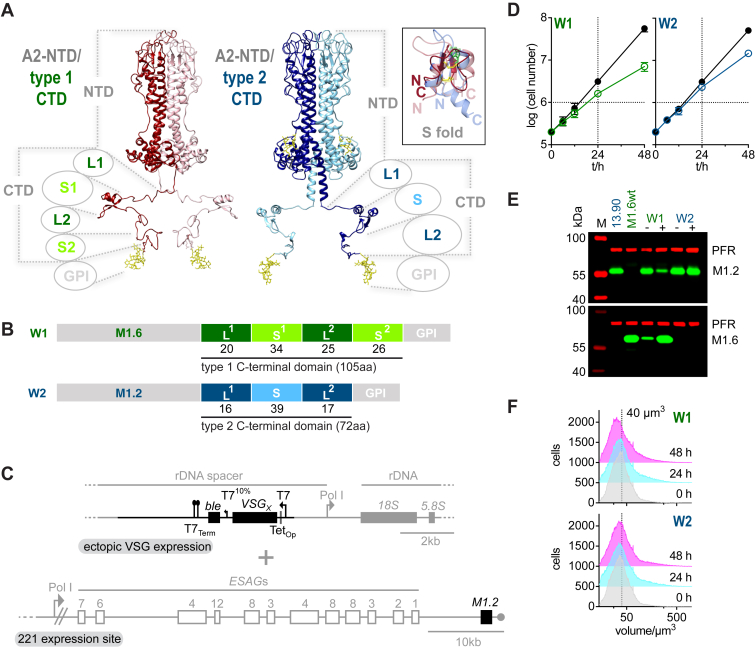
**High**-**level ectopic expression of both MITat1.6 and MITat1.2 support growth of *Trypanosoma brucei***. *A*, the experimentally determined structure models of VSGs containing an A2-type N-terminal domain with either a type 1 C-terminal domain (CTD), or a type 2 CTD (18). Annotations show the N-terminal domain (NTD) and CTD which is composed of two linkers (L1 and L2) and two structured regions (S1 and S2) in type 1 VSGs and two linkers (L1 and L2) and one structured region (S) in type 2 VSGs. N-linked glycans and GPI-gycans are shown in *yellow*. The inset shows the conserved fold of the structured regions of the CTD by means of an overlay of S, S1 and S2 (MITat1.2-S, *blue*, PDB: 1XU6 (27) and ILTat1.24-S1, *light red*, PDB: 2JWG and ILTat1.24-S2, *red*, PDB: 2JWH (28)). Disulfide bonds, *yellow*; conserved aromatic residue, *green*; N and C, N-terminal and C-terminal residue of the structured region. Image prepared with UCSF ChimeraX (53). *B*, schematic overview of the composition of the wild-type VSGs M1.6 (W1) (which has a type 1 C-terminal domain with strong sequence similarity to that of ILTat1.24 (I1.24) (see Fig. S1*A*) and M1.2 (W2). The CTD of W1 consists of two linker regions (L1 and L2) and two structured regions (S1 and S2) and contains 105 amino acids in total. The CTD of W2 consists of two linker regions (L1 and L2) and one structured region S with a total of 72 amino acids. The number of amino acids present in the individual elements of the CTD are given below the schematic image. *C*, ectopic overexpression of VSGs in cells expressing M1.2 from the 221 expression site was achieved by integration of the VSG of interest into the ribosomal DNA (rDNA) spacer region with expression driven by a Tet operator (Tet_Op_) regulated T7 promoter. T7_Term_, T7 terminator; *ble*, *bleomycin* resistance cassette; Pol I, polymerase I promoter, T7^10%^, mutated T7 promoter with 10% activity, *ESAG*, *expression site associated gene*. *D*, ectopically expressed wild-type M1.6 (M1.6wt; W1, *green*) and M1.2 (M1.2wt; W2, *blue*) both support cell growth in a time frame of 48 h. Growth of uninduced cells is shown in *black*, with growth of induced cells shown in *green* for M1.6wt (W1) and in *blue* for M1.2wt (W2). Growth was observed for three independent clones each and is shown as the average with error bars showing the standard deviation. *E*, overexpression of M1.6wt VSG (W1) under the control of a regulated T7 promotor leads to wt level expression and concomitant downregulation of the endogenous VSG M1.2. See also Batram *et al*. (31). Overexpressed M1.2wt VSG (W2) protein also reaches levels found for expression of the endogenous M1.2 in the parental cell line with an RNA dot blot confirming exchange of expression from endogenous to ectopic M1.2wt VSG (see Fig. S1*C*). −, before induction with tetracycline; +, 24 h post induction with tetracycline. M, size standard; PFR, paraflagellar rod - used as a loading control. *F*, graph showing the cell volume distribution of the cell population before (0 h) and 24 h and 48 h post induction of ectopic M1.6wt (W1) and M1.2wt (W2) VSG overexpression. A guideline at 40 μm^3^ volume is displayed to help visualize a shift in the curves from 0 h to 48 h when W1 is overexpressed.

To evaluate the contribution of elements of the VSG CTD that is unique to *T*. *brucei* to VSG function we generated a set of CTD deletion mutants of two wild-type VSGs with the same N-terminal domain classification, A2, but differing in the presence of either one structured region (S) or two structured regions (S1 and S2) - which share a conserved core structure - in their C-terminal domains (Fig. 1, *A* and *B*). These are MITat1.2 (UniProtKB P26332, referred to as M1.2 in the following) which has a type 2 C-terminal domain and MITat1.6 (UniProtKB P26334, referred to as M1.6 in the following) which has a type 1 C-terminal domain. Further sequence information for all wild-type and generated mutant VSGs is provided in the supporting information (Table S1*A*, Fig. S1*A*). Ectopic expression using a tetracycline-inducible expression system with a strong T7 promoter was employed to obtain rapid and robust expression (Fig. 1*C*) (31). Ectopic *VSG* overexpression of a second wild-type *VSG* from a ribosomal DNA spacer region supported cell growth (Figs. 1*D*, S1*B*) and led to downregulation of the native M1.2 VSG resulting in an exchange of the actively expressed major surface protein as determined by Western blotting for induced overexpression of wild-type M1.6 (M1.6wt = W1) (Fig. 1*E*). The exchange of endogenous M1.2 to ectopically expressed wild-type M1.2 (M1.2wt = W2) was confirmed on the mRNA level, based on the fact that the 5′ UTR used for the ectopically expressed M1.2 stemmed from the *GPEET* gene, which codes for one of the major surface proteins of the procylic life cycle stage of *T*. *brucei* (32) (Fig. S1*C*). Western blotting shows an abundant expression of M1.2 following induction of expression of the ectopic VSG (Fig. 1*E*). This system is ideally suited for studying the functionality of VSG mutants for several reasons. First, the reaction on the mRNA-level is almost instantaneous, with the ectopic *VSG* mRNA replacing the native *VSG* mRNA well within one cell cycle. Second, VSG mutants that show any kind of functional defect, will cause a distinct phenotype. As *VSG* exchange on the mRNA level will occur as in wild-type overexpressors, the decrease in endogenous *VSG* mRNA will lead to depletion of its protein product, which - in this scenario - would not be compensated for by production of a functional ectopic VSG. In its extreme, when no or very little protein is produced, this should result in a phenotype resembling VSG ablation that is characterized by the parasites displaying a precise and irreversible pre-cytokinesis cell cycle arrest (11, 12). In contrast, ectopic expression of wild-type (= functional) VSGs will support growth, although partial attenuation of the VSG expression site may occur, which is then accompanied by dormancy, a characteristic, non-lethal accumulation of parasites in the G1-phase of the cell cycle as shown previously, specifically for the overexpression of VSG M1.6 from a M1.2 expressing cell line (31). Measurement of the volume distribution of cells in a population following overexpression of M1.6wt (W1) and M1.2wt (W2) clearly shows a shift to smaller cell volumes in the cell population from 48 h post induction of M1.6wt expression, consistent with G1 accumulation, whereas this is not apparent when overexpressing M1.2wt (Fig. 1*F*). As the effects of dormancy, when present, take hold from around 48 h and any lethal effect from the expression of VSG mutants are expected to arise earlier, the experimental time frame was set to 0 to 48 h post induction of VSG overexpression. Thus, the third advantage of our approach is that the main readouts simply are parasite growth and morphology.

**Figure 2:**
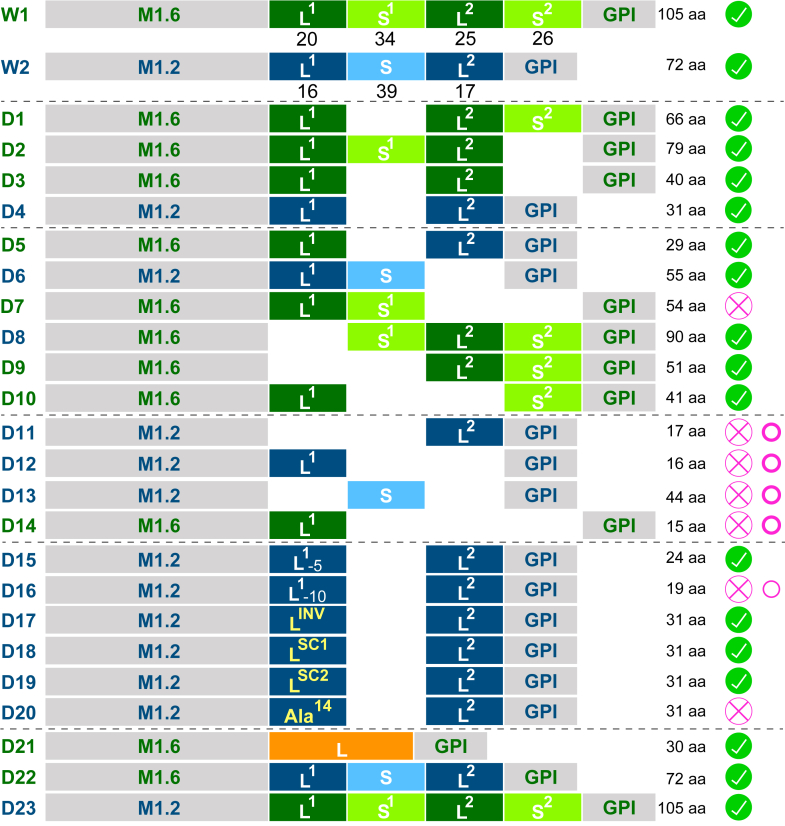
**Schematic overview of the composition of the two wild-type VSGs and their C-terminal domain deletion mutants and VSG chimeras analy**z**ed in this study**. All mutants are based on the wild-type VSGs MITat1.6 (M1.6wt, W1) and MITat1.2 (M1.2wt, W2). Deletion mutants are labelled D1 to D20. Chimeras are labelled D21 to D23. The protein segment origin is indicated by coloring; M1.6: *green*, M1.2: *blue*, *T*. *congolense* VSG BeNat1, *orange*. The total amount of amino acids present in the C-terminal domain of a given construct is given on the *right*. For the wild-type VSGs W1 and W2 the number of amino acids of each linker (L1 and L2) and structured region (S1 and S2 or S) is given below the schematic. The phenotype observed upon overexpression of W1, W2 and D1 to D23 is given with: , supports growth; , does not support growth; , displays strong enlarged FP phenotype; , displays weaker enlarged FP phenotype.

**Figure 3:**
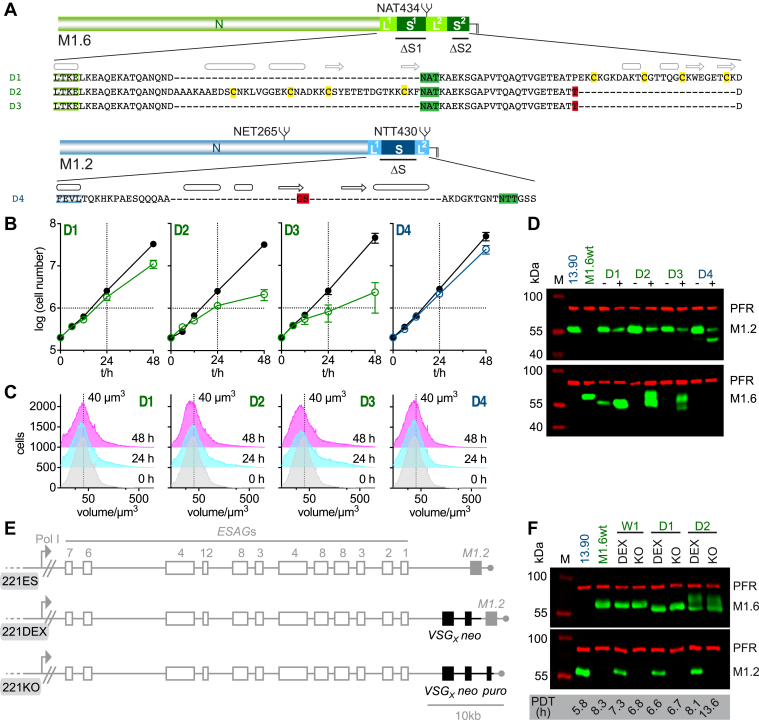
**The structured regions in the C-terminal domains of *T*. *brucei* VSGs are not essential for supporting cell growth**. *A*, amino acid composition of C-terminal domain deletion mutants D1 to D4. D1 to D3 are shown below a schematic depicting the M1.6wt protein composition, D4 is shown under a schematic of the M1.2wt protein. The sequences contain the last four amino acids of the N-terminal and the full sequence of the C-terminal domain. Predicted (*grey*) and known (*black*) secondary structure elements are shown above the amino acid sequences, an arrow for a β-strand and a barrel for an α- or 3_10_-helix. The N-glycosylation site is highlighted in *green*, conserved cysteines in *yellow* and exchanged or added amino acids in *red*. *B*, growth curves of cells harboring mutants D1 to D4. The growth curves are plotted as the average of three independent clones with error bars depicting the standard deviation. Growth curves of the uninduced cells are shown in *black* whereas those following induction of expression of the VSG mutants D1 to D3 are shown in *green* and D4 in *blue*. *C*, The cell volume of mutants D1 to D4 prior to and 24 and 48 h post induction of overexpression of one representative clone each of the respective VSG mutants. As a visual guide a dotted line marks the volume of 40 μm^3^. *D*, a Western blot of whole cell lysates of one representative clone each of mutants D1 to D4 prior to induction of mutant VSG expression (−) and 24 h post induction of expression (+). Samples of the parental M1.2 expressing 13.90 and a wild-type cell line expressing M1.6wt are also shown as controls. M, size standard; PFR, paraflagellar rod - used as a loading control. *E*, schematic showing the composition of the 221 expression site (221ES) and the modified 221ES following generation of cell lines expressing two VSGs from the 221ES (221DEX) and a transgenic cell line expressing only the desired VSG from the 221ES (221KO). Pol I, Polymerase I promoter; *ESAG*, *expression site associated gene*; *neo* and *puro*, *neomycin* and *puromycin* resistance cassettes. *F*, Western blot of whole cell lysates showing one representative clone each of a 221DEX and 221KO cell line of M1.6wt (W1), M1.6ΔS1 (D1) and M1.6ΔS2 (D2) expressing cells. Samples of the M1.2wt expressing parental cell line and of an M1.6wt expressing wt cell line are shown as controls. M, size standard; PFR, paraflagellar rod—served as a loading control. The population doubling time (PDT) of each cell line is listed below the Western blot.

A systematic array of CTD deletion mutants was made based on these two selected wild-type VSGs (Fig. 2, Table S1*B*) with the structured regions of the CTD manipulated first, as they appeared more likely to harbor a functional role than the nonconserved unstructured regions of this domain (Fig. 2, D1-D4 and Fig. 3). As it is possible that S1 and S2 are functionally redundant in VSGs containing two structured regions in their CTD, deletion mutants of M1.6 were generated first, in which either one of the two structured regions, S1 (deletion mutant 1 = D1) or S2 (D2), were deleted (Fig. 3*A*). Induced expression of M1.6ΔS1 (D1) had no negative effect on proliferation compared to cells induced to express the corresponding wild-type M1.6 (W1) (Figs. 3*B* and 1*D*, respectively). Interestingly, overexpression of D1, in contrast to W1, did not lead to an accumulation of cells in G1 by 48 h post-induction (Fig. 3*C*). The M1.6ΔS1 (D1) protein was efficiently expressed, while expression of the native VSG was downregulated (Fig. 3*D*). Thus, the mutant supported cell growth and must therefore be compatible with VSG coat function. Removal of the second structured region S2 to yield M1.6ΔS2 (D2) was conducted in such a way that the C-terminal aspartate of the wild-type protein, to which the GPI-anchor is covalently attached, remained in place (Fig. 3*A*). Following induction of M1.6ΔS2 (D2) expression, the ectopic protein was robustly produced (Fig. 3*D*), and the trypanosomes revealed an overall growth rate that was comparable to that of the wild-type M1.6 control (W1) (Figs. 3*B* and 1*D*, respectively). Thus, deletion of S2 was also possible. In contrast to D1, overexpression of D2 did lead to an accumulation of cells in G1 as observed for M1.6wt (Figs. 3*C* and 1*F*, respectively).

The finding that M1.6 VSGs lacking either their S1 or S2 region were successfully incorporated into the trypanosome cell surface coat could, in fact, point to a functional redundancy of the two structured regions. Therefore, a mutant was constructed in which both structured regions were removed. Cells expressing M1.6ΔS1ΔS2 (D3) displayed cell growth and a cell volume distribution that was comparable to that of cells expressing the single deletion mutant D2 or the wild-type M1.6 (W1) on their cell surface (Fig. 3, *A*–*D* and Fig. 1, *D* and *F*, respectively). This suggested that neither of the structured regions in the C-terminal domain of a *T*. *brucei* VSG is required for the structural integrity of the protein, its routing to the cell surface, or formation of a variant surface glycoprotein coat. To corroborate this counterintuitive finding, the single structured region S of the C-terminal domain of M1.2 was deleted. Following induction of ectopic expression of M1.2ΔS (D4), cell growth was the same as observed after induction of the wild-type VSG M1.2 (W2) (Figs. 3*B* and 1*D*, respectively).

We next tested whether D1 and D2 alone can form a functional VSG coat. For this, the mutant genes were integrated into the active M1.2 expression site, with subsequent deletion of the native M1.2 VSG gene (Fig. 3*E*). Successful generation of trypanosomes expressing only the mutant VSG D1 or D2 was confirmed by Western blotting, and population doubling times for these cell lines were determined (Fig. 3*F*). As trypanosomes stall in growth when coat formation fails, neither S1 nor S2 of M1.6 appears to be essential for VSG coat formation.

Therefore, we conclude that the conserved, structured regions in the C-terminal domain of *T*. *brucei* VSGs are not required for the formation of the parasite cell surface coat.

### The composition of the GPI-anchoring region is flexible though functionally optimized

**Figure 4:**
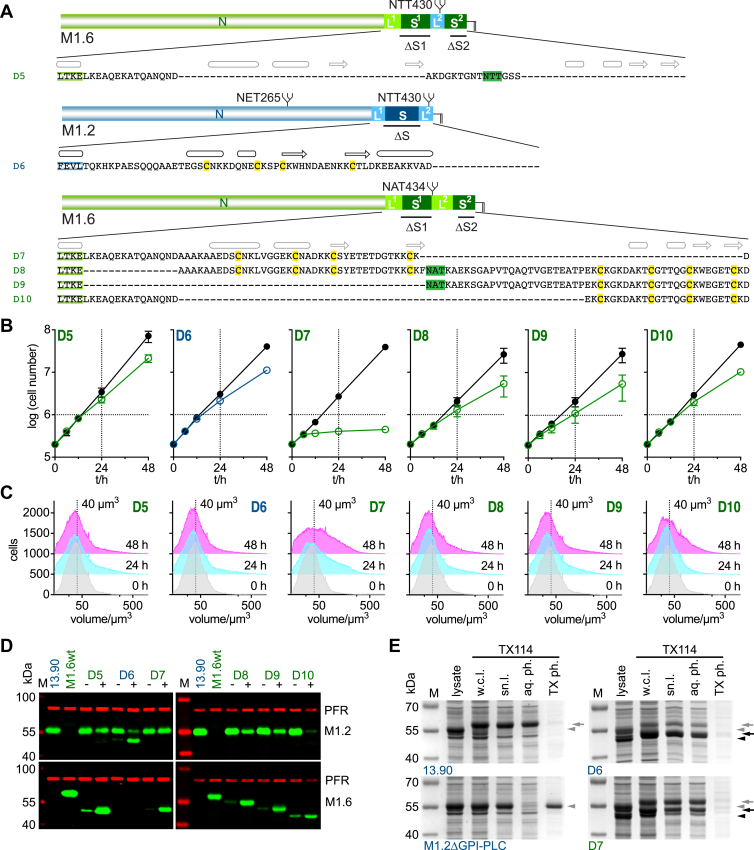
**The GPI-anchoring regions of VSGs, though adapted, are flexible in composition and several deletions in this region are tolerated without impacting on successful GPI-anchoring of the mutant VSG**. *A*, amino acid sequences of C-terminal domain mutants D5 to D10. D5 is shown below a schematic chimeric VSG in which the L2 of M1.6 is preplaced by that of M1.2 (shown in *blue*). Deletion mutant D6 is shown below a schematic of the M1.2wt protein and deletion mutants D7 to D10 are shown below a schematic of the M1.6wt protein. The sequences contain the last four amino acids of the N-terminal domain (*green*: M1.6 N, *blue*: M1.2 N) in addition to the sequence of the respective C-terminal domain. Predicted (*grey*) or known (*black*) secondary structure elements are shown above the amino acid sequences, an arrow for a β-strand and a barrel for an α- or 3_10_-helix. N-glycosylation sites are highlighted in *green* and conserved cysteines in *yellow*. *B*, growth curves of cells transfected with mutants D5 to D10. The growth curves are plotted as the average of three independent clones with error bars depicting the standard deviation. The growth curves of uninduced cells are shown in *black* whereas the growth curves following induction of expression of the VSG mutants D5 and D7 to D10 are shown in *green* and D6 in *blue*. *C*, the cell volume of mutants D5 to D10 prior to and 24 and 48 h post induction of overexpression of one representative clone each of the respective VSG mutants. As a visual guide a dotted line marks the volume of 40 μm^3^. *D*, a Western blot of whole cell lysates of one representative clone each of mutants D5 to D10 prior to induction of mutant VSG expression (−) and 24 h post induction of expression (+). Samples of the parental M1.2 expressing 13.90 and a wild-type cell line expressing M1.6wt are shown as controls. M, size standard; PFR, paraflagellar rod - used as a loading control. *E*, Coomassie stained gels showing whole cell lysates and samples taken during Triton X-114 (TX114) cell lysis and phase separation of the parental M1.2 expressing 13.90 cell line, an M1.2 expressing GPI-PLC knock-out cell line (M1.2ΔGPI-PLC) (54), and D6 and D7 expressing cells following 24 h of tetracycline induction. M, size standard; lysate, whole cell lysate generated by addition of protein sample buffer to harvested cells; w.c.l., whole cell lysate generated by addition of TX114; sn.l., supernatant after centrifugation of the TX114 whole cell lysate; aq. ph., aqueous phase after phase separation; TX ph., TX114 phase after phase separation. *Gray arrowheads*, M1.2 mfVSG; *gray arrows*, M1.2 sVSG; *black arrowheads*, D6 or D7 mfVSG; *black arrows*, D6 or D7 sVSG.

In both D2 and D3, the L2 linker region, which normally links S1 to S2, has to function as the GPI-anchoring site. As shown for D2, removal of S2 led to an increase in population doubling time when constitutively expressed; however, the cells were viable. We propose that L2 can function as the GPI-anchoring site, albeit less efficiently, which is reflected in the slower growth of the parasites. The trypanosomes do not reveal any apparent phenotype besides slowed proliferation, which may mean that the production of VSG and hence the cell cycle is slowed. That a flexible linker can, in principle, evolve to be a GPI-anchoring structure is shown by type 2 VSGs, which feature an L2-linker on their C-terminal end (Fig. 2, W2). Thus, we used this “natural” GPI-anchoring structure to replace the “non-natural” linker L2 of D3. This chimeric VSG mutant D5 consisted of the N-terminal domain and linker L1 of VSG M1.6, followed by linker L2 from VSG M1.2 (Fig. 4*A*). Cells induced to express this chimeric VSG, as could be expected, were not impaired in growth, which resembled the growth observed when overexpressing W2 more closely than that following induced expression of D3 or W1 (Figs. 3 and 4 or 1D, respectively). No apparent change in average cell volume of the population occurred within 48 h, again more closely resembling the observations made for W2 than those for D3 or W1 expression (Figures 1F, respectively). High-level expression of the mutant was verified by Western blotting (Fig. 4*D*).

M1.2ΔL2 (D6), a VSG M1.2 mutant lacking its linker L2, which is GPI proximal in the wild-type VSG, was expressed well and lead to no apparent phenotypic changes suggesting that the single structured region S can assume a GPI-anchoring role (Fig. 4, *A*–*D*). In stark contrast, a structurally similar mutant of VSG M1.6, M1.6ΔL2ΔS2 (D7), showed a marked growth phenotype, with growth stalling from 6 h post induction of expression (Fig. 4, *A* and *B*). The average cell volume of the population clearly increased upon induction of expression (24 h and 48 h timepoints) with expression of the mutant clearly detectable on a Western blot (Fig. 4, *C* and *D*). In this recombinant protein, the second motif (L2 and S2) of the CTD was completely removed, forcing GPI-addition to the structured region S1. The actual presence of a GPI-anchor for both D6 and D7 was shown by generating cell lysates under conditions that suppressed or allowed GPI-PLC activity to yield the intact membrane form VSG (mfVSG) or the cleaved soluble VSG (sVSG) of higher apparent molecular weight, respectively. The phase separation properties of Triton X-114 were used to show that the sVSG, lacking the lipid part of the GPI-anchor, separated into the aqueous phase (Fig. 4*E*) (33, 34). Thus, although the structured regions of different VSG CTDs share a conserved core, subtle differences may affect the functionality of the GPI-anchored protein, probably due to steric effects. A notable difference between S of M1.2 and S1 of M1.6 is the presence of a C-terminal α-helix in addition to the conserved core structure in the former (Fig. 1*A* (inset), Fig. 4*A*). This supports the notion that the region upstream of the GPI-addition site can vary significantly within a framework that supports VSG anchoring and coat formation. Furthermore, it is reasonable to assume that the different growth rates observed in different trypanosome strains may depend on the VSG expressed and more specifically on the nature of the C-terminal domain.

### The interdomain linker L1 is not an essential component of VSGs

Thus far, the data have suggested that VSGs do not require the evolutionarily conserved structured regions in their C-terminal domain, but that a functionally optimized sequence is required just upstream of the GPI-addition site. This can be either a structured region or a flexible linker region. In addition to this GPI-attachment region, all the mutants generated so far (D1-D7) have retained the linker L1 in their C-terminal domain, which in all known VSGs connects the N-terminal domain to its first structured region. To test whether this linker L1 is essential, two L1 deletion mutants, both based on VSG M1.6wt, were generated. In M1.6ΔL1 (D8), only L1 was removed, whereas in M1.6ΔL1ΔS1 (D9) the complete upper CTD module, consisting of L1 and S1, was deleted (Fig. 4*A*). Both mutants supported cell growth in the 48 h following induction of expression, showing that L1 is not essential as such (Fig. 4*B*). This suggests that the flexible linker L2 that is present in D9 can assume the role of L1. Thus, neither the structured regions nor the flexible interdomain-linker L1 are essential building blocks of the C-terminal domain.

### A *T*. *brucei* VSG requires an N-terminal domain, a linker region, and a GPI-anchoring region to support cell viability

**Figure 5:**
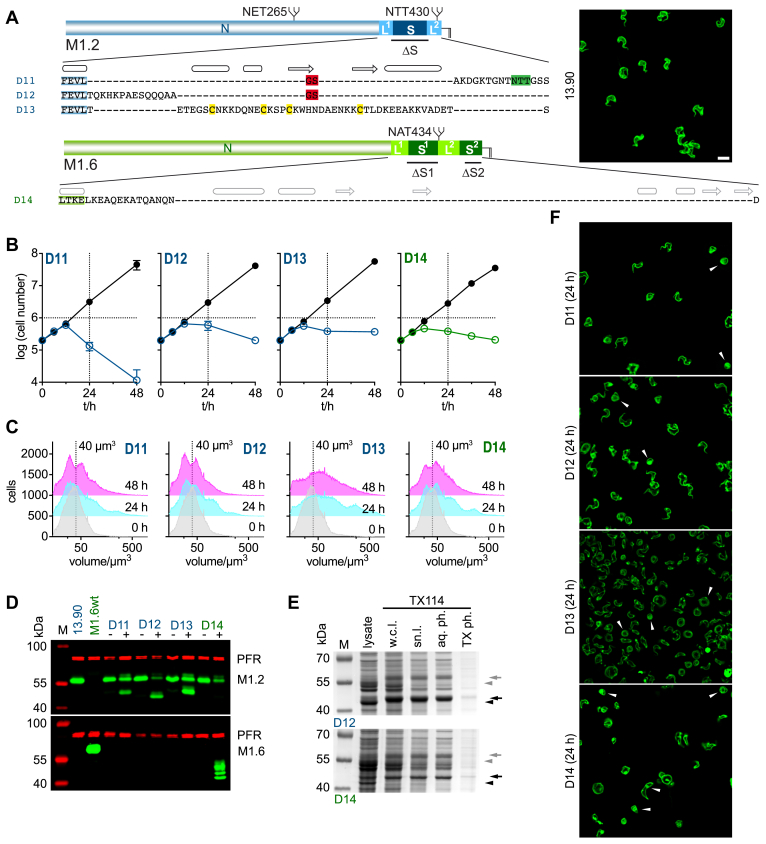
**The C-terminal domain of a *T*. *brucei* VSG needs to contain a linker region in addition to a GPI-anchoring region**. *A*, amino acid composition of C-terminal domain deletion mutants D11 to D14. D11 to D13 are shown below a schematic depiction of the M1.2wt protein composition, D14 is shown under a schematic of the M1.6wt protein. The sequences contain the last four amino acids of the N-terminal domain (*blue*: M1.2 N, *green*: M1.6 N) and the full sequence of the C-terminal domain. Predicted (*grey*) or known (*black*) secondary structure elements are shown above the amino acid sequences, an arrow for a β-stand and a barrel for an α- or 3_10_-helix. The N-glycosylation site, where present, is highlighted in *green*, conserved cysteines in *yellow* and added amino acids in *red*. Inset, microscopy image showing NHS-Atto488 (*green*) surface-stained parental 13.90 cells that express M1.2. Scale bar 10 μm. *B*, growth curves of cells harboring mutants D11 to D14. The growth curves are plotted as the average of three independent clones with error bars depicting the standard deviation. Growth curves of the uninduced cells are shown in *black* whereas the growth curves following induction of expression of the VSG mutants D11 to D13 are shown in *blue* and D14 in *green*. *C*, the cell volume of mutants D11 to D14 prior to and 24 and 48 h post induction of overexpression of the respective VSG mutant in one representative clone each. As a visual guide a dotted line marks the volume of 40 μm^3^. *D*, a Western blot of whole cell lysates of one representative clone each of mutants D11 to D14 prior to induction of mutant VSG expression (−) and 24 h post induction of expression (+). Samples of the parental M1.2 expressing 13.90 and a wild-type cell line expressing M1.6wt are shown as controls. M, size standard; PFR, paraflagellar rod - used as a loading control. *E*, Coomassie stained gels showing whole cell lysates and samples taken during Triton X-114 (TX114) cell lysis and phase separation of D12 and D14 expressing cells following 24 h of tetracycline induction. For the controls of parental M1.2 expressing 13.90 cell line and an M1.2 expressing GPI-PLC knock-out cell line see Fig. 5*E*. M, size standard; lysate, whole cell lysate generated by addition of protein sample buffer to harvested cells; w.c.l., whole cell lysate generated by addition of TX114; sn.l., supernatant after centrifugation of the TX114 whole cell lysate; aq. ph., aqueous phase after phase separation; TX ph., TX114 phase after phase separation. *Grey arrowheads*, M1.2 mfVSG; *gray arrows*, M1.2 sVSG; *black arrowheads*, D12 or D14 mfVSG; black arrows, D12 or D14 sVSG. As D14 displays a heterogeneous protein pattern, only the shift of the lower band is highlighted in the gel image. *F*, microscopy images showing NHS-Atto488 (*green*) surface-stained cells induced to express VSG mutants D11-D14 for 24 h. Selected cells displaying enlarged flagellar pockets are marked with an *arrowhead*. Scale bar as shown in *A*.

**Figure 6:**
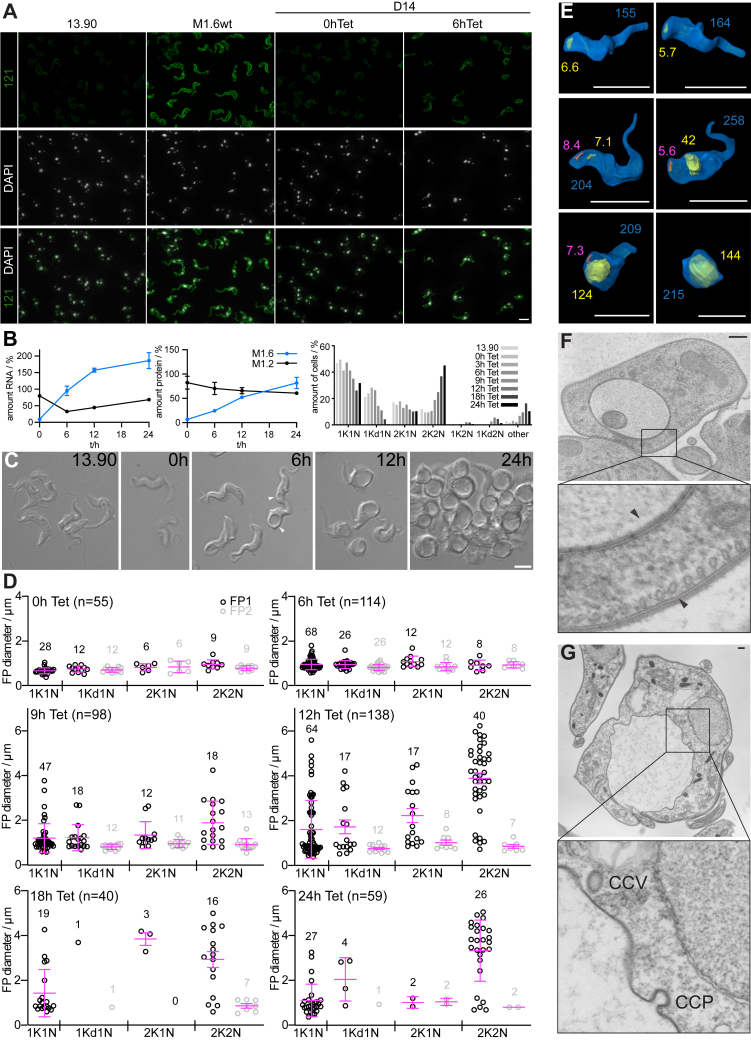
**The non-functional M1.6 deletion mutant D14 is abundantly expressed and located on the cell surface**. *A*, the expressed M1.6 deletion mutant D14 is routed to the cell surface. Indirect immunofluorescence analysis of cells before (0 h) and 6 h after induction of D14 expression using a polyclonal anti-MITat1.6 antibody. The parental 13.90 cell line, expressing VSG M1.2, and a VSG M1.6 expressing wild-type cell line (M1.6wt) show specificity of the antibodies used. The VSG is shown in *green*, DAPI stained nuclei and kinetoplasts in *grey*. Scale bar 10 μm. *B*, M1.6 and M1.2 mRNA (*left*) and protein levels (*middle*) during the course of tetracycline-induced expression of mutant D14. VSG mRNA and protein amounts were quantified from three independent clones each using an RNA and protein dot blot, respectively, are normalized to tubulin (mRNA) or PFR (protein), respectively, and are plotted as averages with error bars representing the standard deviation. Cell cycle analysis of the parental 13.90 cell line and the D14 expressing cell line (*right*), before and at different time points after induction of expression. Cells were chemically fixed and stained with DAPI for visualization of nuclei (N) and kinetoplasts (K) by light microscopy. At least 300 cells were analyzed for each time point of D14 expressing cells; n = 257 for the parental cell line. Cells were grouped into 1K1N for cells displaying 1 kinetoplast and 1 nucleus (G1-phase), 1Kd1N for cells displaying a dividing kinetoplast, 2K1N for cell in G2/M-phase and 2K2N for post-mitotic/pre-cytokinesis cells. Cells displaying unusual kinetoplast and nuclei configurations are labelled with 2K2N, 1Kd2N or “other.” *C*, Light microscopy images (DIC) of live cells during the course of tetracycline-induced D14 expression. From *left* to *right*, the parental 13.90 cell line employed in this study and cells prior to induction of D14 expression (0 h) and 6, 12, and 24 h post induction of D14 expression. Enlarged flagellar pockets in cells 6 h post induction of D14 expression are indicated by *white arrowheads*. Scale bar 5 μm. *D*, graphs showing the increase in flagellar pocket size following induction of expression of D14. The flagellar pocket sizes are plotted as individual data points and are shown as equivalent spherical diameters grouped by the cell cycle stage of the cell with old (*black*) and new (*grey*) flagellar pocket sizes clearly marked. The mean and standard deviation of flagellar pocket sizes in the groups are shown in *magenta*. The total number of cells analyzed is shown in each graph along with the total number of cells analyzed for each cell cycle stage. Cells with aberrant cell cycle configuration were omitted. *E*, enlarged flagellar pockets can reach surface areas in the range of the cell surface area of a G1-phase cell. The cell surface (*blue*) as well as the flagellar pockets (*yellow*, single or old flagellar pocket, *magenta*, new flagellar pocket) were modelled from microscopy data of surface stained (NHS-Atto488), chemically fixed cells. The figure shows representative cells displaying a range of flagellar pocket sizes. Numbers indicate membrane areas for the single or old flagellar pocket (*yellow*), the new flagellar pocket (*magenta*) and the cell surface (*blue*) in μm^2^. Scale bars 10 μm. *F*, electron micrograph of a high pressure frozen and Epon embedded cell displaying an enlarged flagellar pocket. The enlarged section shows the presence of the electron dense VSG surface coat (*arrow heads*). Scale bar 250 nm. *G*, electron micrograph of a chemically fixed and Epon embedded cell displaying an enlarged flagellar pocket. The enlarged section shows the presence of a clathrin coated pit (CCP) and a clathrin coated vesicle (CCV). Scale bar 250 nm.

The deletion mutants D8 and D9, with D10 acting as a control, suggested that a naturally evolved L1 is not essential for VSG function. Furthermore, the linker L2 of M1.6 can deputize as a GPI-addition sequence (Fig. 3; D2, D3). Thus, we surmised that a flexible linker might be all that is required to form the C-terminal end of a VSG and that the CTD is not relevant for coat formation at all. To test this, mutant M1.2ΔL1ΔS (Fig. 5*A*; D11) was generated. This transgene contains the N-terminal domain and the linker region L2 to which the GPI-anchor is attached. This mutant was not functional, as apparent from the observed reduction in cell number from 12 h post induction of expression (Fig. 5*B*). In addition, a shift to a larger average cell volume in the cell population could be detected (Fig. 5*C*). This was despite efficient protein expression of D11 (Fig. 5*D*). The same lethal phenotype was observed upon deletion of both S and L2 from M1.2 (Fig. 5, *A*–*D*; D12), leaving only L1 in the CTD of the protein. Deletion of both L1 and L2 which leaves only S in the CTD of M1.2 also resulted in severe enlargement of the cells (Fig. 5, *A*–*D*; D13). Another mutant comparable to D11 and D12 was based on VSG M1.6 with S1, L2 and S2 removed while ensuring the C-terminal amino acid that is used for GPI-anchor attachment was left in place (Fig. 5*A*; M1.6Δ(S1-S2), D14). Expression of this mutant was also abundant and led to an increase in cell volume (Fig. 5, *B*–*D*). GPI anchoring was confirmed for both D12 and D14, which contain altered protein sequences upstream of the ω-site (Fig. 5*E*). The observed increase in average cell volume was due to the parasites displaying a marked increase in flagellar pocket size, leading to rounding up of cells and eventual cell death (Fig. 5*F*). Immunofluorescence analysis clearly showed that the GPI-anchored, yet non-functional mutant protein D14 was correctly routed to the cell surface of the trypanosome (Fig. 6*A*). In accordance with previous wild-type VSG overexpression experiments (31), the ectopic expression of the mutant VSG caused a rapid decrease of the native VSG mRNA (Fig. 6*B*). This, however, was followed by only a mild loss of wild-type protein (Fig. 6*B*). This lack of downregulation of endogenous VSG protein in response to overexpression of the ectopic VSG can be explained by the rapid cell cycle arrest in G2 (Fig. 6*B*) and the relatively high stability of VSG proteins (35). In the arrested trypanosomes no VSG dilution through cell division occurred. Evidently, the plasma membrane was still being produced, but as the cell does not initiate cytokinesis, the newly synthesized membrane was retained in the flagellar pocket (Fig. 6, *C*–*G*). This is the only area of the plasma membrane that is not tethered to the cytoskeleton and that can grow in size. In fact, we measured the increase in flagellar pocket area, which was equivalent to one complete trypanosome cell surface (Fig. 6*E*). Electron micrographs of cells expressing the mutant confirm the presence of a dense protein coat, both on the outer cell surface and on the surface of the flagellar pocket membrane (Fig. 6*F*). In addition, the presence of clathrin-coated pits and clathrin-coated vesicles shows that endocytic uptake is still occurring (Fig. 6*G*).

We were left with the question of how to explain that expression of VSGs with a CTD reduced to just one linker region is lethal. In both wild-type VSGs, linker L1 attaches the N-terminal domain to the CTD, and linker L2 functions well as a GPI-addition sequence even in M1.6wt, where the C-terminal S2 naturally harbors the GPI-attachment site (Fig. 3; D2). When both linkers, L1 and L2, were combined to form the CTD (Figs 3 and 4; D3-D5), the mutant VSGs were functional in the surface coat. Thus, both attachment functions can be conferred by these flexible linkers. A straightforward explanation for the observed data was that one linker alone simply was too short to be functional.

### *T*. *brucei* VSG C-terminal domains have a minimum size requirement

**Figure 7:**
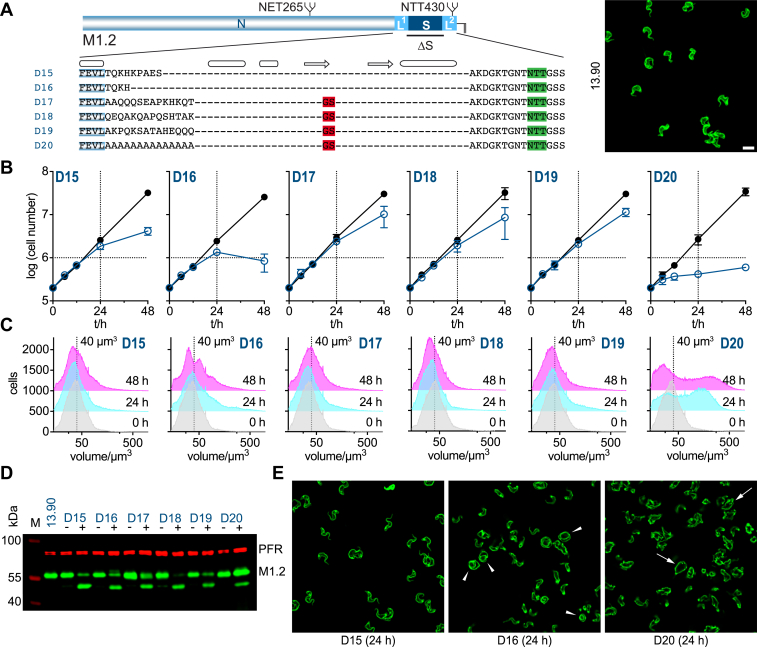
**The C-terminal domain of *T*. *brucei* VSGs requires a minimal size to support cell growth whilst the composition of the linker L1 is flexible**. *A*, amino acid sequences of C-terminal domain mutants D15 to D20 shown below a schematic of the M1.2wt protein. The sequences contain the last four amino acids of the N-terminal of M1.2 (blue) in addition to the sequence of the respective C-terminal domain. Known secondary structure elements are shown above the amino acid sequences, an arrow for a β-strand and a barrel for an α-helix. N-glycosylation sites are highlighted in *green* and added amino acids in *red*. Inset, microscopy image displaying NHS-Atto488 (*green*) surface-stained parental 13.90 cells that express M1.2. Scale bar 10 μm (image reused from Fig. 5*A*). *B*, growth curves of cells transfected with mutants D15 to D20. The growth curves are plotted as the average of three independent clones with error bars depicting the standard deviation. The growth curves of uninduced cells are shown in *black* whereas the growth curves following induction of expression of the VSG mutants are shown in *blue*. *C*, the cell volume of mutants D15 to D20 prior to and 24 and 48 h post induction of overexpression of one representative clone each of the respective VSG mutants. As a visual guide a dotted line marks the volume of 40 μm^3^. *D*, Western blot of whole cell lysates of one representative clone each of mutants D15 to D20 prior to induction of mutant VSG expression (−) and 24 h post induction of expression (+). A sample of the parental M1.2 expressing 13.90 cell line is shown as a control. M, size standard; PFR, paraflagellar rod - used as a loading control. *E*, Microscopy images displaying NHS-Atto488 (*green*) surface-stained cells induced to overexpress VSG mutants D15, D16 and D20 for 24 h. Selected cells with enlarged flagellar pockets in D16 expressing cells are highlighted with an *arrowhead*, selected cells displaying a cytokinesis defect in D20 overexpressing cells are marked with an arrow. Scale bar as shown in *A*.

Results from the VSG deletion mutants D11, D12, and D14 (Fig. 5) suggest that the C-terminal domain might have a minimum size requirement. These mutants retained less than 20 amino acids of the C-terminal domain linker. In addition, the C-terminal domain must be flexible, as mutant D13, which leaves the 44 amino acid containing structured region S, had impaired functionality. Thus, a minimum size and flexibility are required. To test this possibility, two mutants of VSG M1.2 were constructed, in which S was deleted and L1 was successively shortened, generating M1.2L1^-5^ΔS (D15) and M1.2L1^-10^ΔS (D16) (Fig. 7). Shortening of L1 by 5 amino acids (D15) supported cell growth in a timeframe of 48 h post induction of expression, without any apparent effect on flagellar pocket size, whereas shortening of L1 by 10 amino acids (D16) yielded cells displaying enlarged flagellar pockets. These experiments are suggestive of the existence of a minimal *T*. *brucei* VSG, which is composed of an N-terminal domain, a linker region L1 requiring a minimum length of about 30 amino acids, and a region optimized for GPI-anchor attachment, which can be either flexible (*e*.*g*. L2) or structured (*e*.*g*. S2). As linker L2 could replace L1 in the mutant M1.6ΔL1ΔS (Fig. 4; D9), we tested whether there were any constraints on amino acid composition for the linker L1. A set of mutants was generated based on the VSG deletion mutant M1.2ΔS (Fig. 2; D4). In M1.2L1^inv^ΔS (Fig. 7*A*; D17), the amino acid sequence in L1 was inverted. Expression of this construct had no adverse effect on cell growth (Fig. 7*B*). Likewise, M1.2L1^SC1^ΔS (D18) and M1.2L1^SC2^ΔS (D19) in which the amino acid sequence of L1 was randomly scrambled, both supported cell proliferation with the protein efficiently expressed (Fig. 7, *A*–*D*). While the cell volume distribution appeared to stay constant following induction of expression of D17 and D19, a clear shift to smaller average volumes could be observed for D18, suggesting an accumulation of cells in G1 (Fig. 7*C*). Hence, while D17 and D19 behave similar to D4 and W2, D18 behaves more like W1. This is also supported by their population growth when observed over a time period of 168 h (Fig. S2). Replacement of all amino acids in L1 by alanine, a common small amino acid substitute that maintains main-chain conformation, M1.2L1^Ala14^ΔS (Fig. 7*A*; D20), yielded a marked growth phenotype with cell proliferation severely affected from 12 h post induction of expression of the mutant. However, cells did not display an increase in FP size as observed for mutants D11-D14 and D16, but rather a cytokinesis defect with aberrant cells being formed (Fig. 7*E*). This result shows that size, flexibility, and some degree of amino acid diversity are required for the minimal C-terminal domain of *T*. *brucei* VSGs.

### The minimal C-terminal domain of a *T*. *congolense* VSG can function in *T*. *brucei* VSGs

*T*. *brucei* VSGs with a type 4 C-terminal domain represent one of the two theoretically possible versions of a “minimal” VSG, as they contain only a linker L1 and a structured region S to which the GPI-anchor is covalently attached (20). The second possible version, VSGs with a C-terminal domain containing only linkers L1 and L2, has never been reported for *T*. *brucei* VSGs, although our mutagenesis study has shown that this combination can be functional.

**Figure 8:**
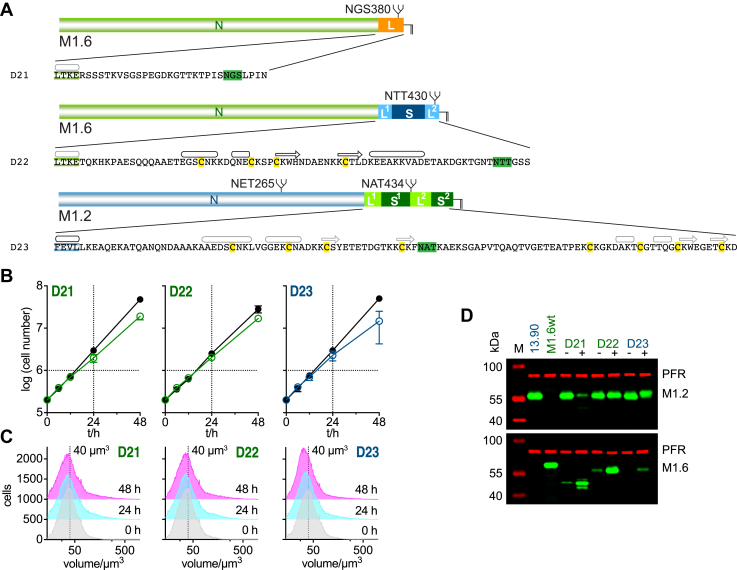
**Chimeric VSGs are readily expressed with the C-terminal domain influencing cell growth**. *A*, amino acid sequences of C-terminal domain mutants D21 to D23 each shown below a schematic of the respective chimeric VSG. The sequences contain the last four amino acids of the N-terminal (*green*: M1.6 N, *blue*: M1.2 N) in addition to the sequence of the respective C-terminal domain. Predicted (*grey*) or known (*black*) secondary structure elements are shown above the amino acid sequences, an arrow for a β-strand and a barrel for an α- or 3_10_-helix. N-glycosylation sites are highlighted in *green* and conserved cysteines in *yellow*. *B*, growth curves of cells transfected with mutants D21 to D23. The growth curves are plotted as the average of three independent clones with error bars depicting the standard deviation. The growth curves of uninduced cells are shown in *black* whereas the growth curves following induction of expression of the VSG mutants are shown in *green* (M1.6 NTD) or *blue* (M1.2 NTD). *C*, the cell volume of mutants D21 to D23 prior to and 24 and 48 h post induction of overexpression of one representative clone each of the respective VSG mutants. As a visual guide a dotted line marks the volume of 40 μm^3^. *D*, a Western blot of whole cell lysates of one representative clone each of mutants D21 to D23 prior to induction of mutant VSG expression (−) and 24 h post induction of expression (+). Samples of the parental M1.2 expressing 13.90 and a wild-type cell line expressing M1.6wt are shown as controls. M, size standard; PFR, paraflagellar rod—used as a loading control.

Interestingly, VSGs from another African trypanosome species, *T*. *congolense*, seem to have exclusively evolved such an unstructured and very short C-terminal domain. *T*. *congolense* VSGs are composed of an N-terminal domain that shares many characteristics with the equivalent domain in *T*. *brucei* VSGs, but they have a very different C-terminal domain, as they do not possess the conserved cysteine residues which form a central part of the structured regions in the *T*. *brucei* VSG C-terminal domains (36). Our data suggests that these cysteines are dispensable also in *T*. *brucei* CTDs, which prompted us to ask if the short C-terminal region of a *T*. *congolense* VSG would support cell growth of *T*. *brucei* when combined with a *T*. *brucei* VSG N-terminal domain. The number of amino acids present in the *T*. *congolense* VSG C-terminal extension is similar to that found in the combined L1 and L2 in the minimal VSG M1.2ΔS (D4) generated from M1.2wt. Can the naturally short CTD of a *T*. *congolense* VSG, therefore, replace the C-terminal domain of a *T*. *brucei* VSG? To test this, a chimeric VSG composed of the M1.6 N-terminal domain and the C-terminal domain of the *T*. *congolense* VSG BeNat1 (29) was generated (Fig. 8*A*; D21). This chimeric VSG was successfully expressed and supported cell proliferation (Fig. 8, *B*–*D*). Hence, the conserved structured regions present in the C-terminal domain of *T*. *brucei* VSGs are not essential for successful coat formation in this species of African trypanosomes.

### The C-terminal domain of a VSG modulates cell growth

Our finding that the structured regions of *T*. *brucei* VSG CTDs are evolutionarily highly conserved, but not essential for coat formation prompted us to think about functions of the CTD that were more subtle, and not directly related to cell survival by formation of an operative surface coat. We had previously observed that the expression of different VSGs in the same trypanosome strain led to characteristic changes in the population doubling times. When some VSGs were expressed, the parasites doubled every 6 h, but doubling times could increase to as much as 8 h when other VSGs were expressed. It has been suggested that this phenomenon might be explained by the overall VSG protein size (37). Our results, however, suggest that the architecture of the C-terminal domain may influence cell growth as seen when comparing growth following expression of same size VSG mutants D4 and D17-D19. To further test this, we generated a chimeric VSG mutant consisting of an M1.6 N-terminal domain and an M1.2 C-terminal domain (Fig. 8*A*; D22). This chimeric VSG was successfully expressed in *T*. *brucei* (Fig. 8*D*). Comparison of population doubling times when expressing wild-type M1.2 and M1.6 and the chimeric M1.6N-M1.2 C VSG showed that growth and average cell volume distribution following expression of the chimeric VSG more closely resembled that found in cells following expression of wild-type M1.2 (W2) (Fig. 8, *B* and *C* and Fig. 1, *D* and *F*). An inverse chimeric VSG containing the N-terminal domain of M1.2 and C-terminal domain of M1.6 (Fig. 8*A*; D23) was expressed and supported cell growth. While growth attenuation was not as strong as for M1.6wt (W1) a slight slowing in growth compared to M1.2wt (W2) was observed (Fig. 8, *B*–*D* and Fig. 1, *D*–*F*). Thus, the C-terminal domain appears to influence VSG-dependent growth control in *T*. *brucei*. The VSG C-terminal domain might therefore modulate population growth in bloodstream stage trypanosomes, a feature that is likely to be non-essential *in vitro*, but might become relevant when in the mammalian host, a scenario that is testable but clearly out of scope of the present study.

## Discussion

Many protozoans have evolved surface coats as a virulence factor in order to be able to establish infections in their hosts. One of the best analyzed surface coats in terms of cell biology and structure is that of the African trypanosome *T*. *brucei*. *T*. *brucei* is covered by a dense surface coat of a GPI-anchored VSG. Other African trypanosomes such as *T*. *congolense* and *T*. *vivax* also express VSG coats on their cell surface. The common function of this coat is to protect the parasite from destruction by its mammalian host. Much of what we know about the VSG repertoire, its regulation and the architecture of the surface coat, which lies at the parasite-host interface, is based solely on studies of *T*. *brucei*. Certain aspects of coat biology have evolved differently in other African trypanosomes, however (reviewed in (38)). One such difference is the domain architecture of the VSG, with the C-terminal domain composition of *T*. *brucei* VSGs being unique to this species (30). Structural analyses together with single molecule diffusion measurements suggest that this C-terminal domain supports VSG flexibility by allowing it to adopt two distinct conformations which might support coat integrity during, for instance, antigenic variation and cell division (18). Although conserved in all known functional *T*. *brucei* VSGs (3), we show here that the structured regions are not essential for coat formation, and replacement of a *T*. *brucei* VSG C-terminal domain by that of a *T*. *congolense* VSG C-terminal supports cell proliferation. The minimal composition of a VSG requires an N-terminal domain and a C-terminal domain with a linker L1 that connects to the N-terminal domain, and a GPI-anchoring region that can be flexible (L2) or structured (S2). Although the exact composition of these modules is diverse, they appear to be optimized for their function. Deletion mutants in which structured regions that do not usually function as GPI-linking regions were placed at the C-terminus could, but did not always, support cell viability. This, on the one hand, underlines the modular composition of the VSG C-terminal domain, an important facet in light of expression of mosaic VSGs during the course of an infection (6, 7, 22, 24, 39, 40, 41), but importantly, on the other hand, also suggests that not all seemingly intact VSGs found in the genome will in fact be functional VSGs.

We have previously shown that the C-terminal domain of VSGs can adopt different conformations, thereby changing the height and the footprint of the VSG (18). This surprising flexibility could play an essential role when freely diffusing VSGs encounter invariant surface proteins, cushioning the influence of obstacles. It was also speculated that the IgM and IgG-mediated tethering of two VSGs could lead to protein bending that could be compensated by the suspension-function of the CTD. Furthermore, during antigenic variation, the flexibility of the CTD could allow accommodation of differently sized VSGs on the cell surface, thereby always guaranteeing complete coverage of the plasma membrane. In the present study, we have shown that a minimal CTD of about 30 amino acids suffices to support formation of a functional VSG coat. The solution structures of VSG CTDs (18, 27, 28) suggest that the structured regions may function as compact hinges during conformational switching. The combination of two flexible linkers (D5; 29 amino acids) produces a CTD that could extend similarly far above the plasma membrane as a type 2 CTD, which is likewise the case if a linker and a more compact structured region are combined (D6; 55 amino acids). When the linker length is reduced further, the CTD function is lost. Thus, it appears that just size and flexibility matter. This comes as a surprise, as the CTD structured regions are highly conserved in all *T*. *brucei* VSGs (27, 28). In contrast, neither *T*. *congolense* nor *T*. *vivax* VSGs appear to possess a C-terminal domain of similar composition (36, 42). Interestingly, our data show that a *T*. *brucei* VSG also functions with a C-terminus of a *T*. *congolense* VSG. The *T*. *congolense* CTD is about the same size as the minimal *T*. *brucei* CTDs that were iteratively generated during the present study. Thus, *T*. *congolense* VSGs possess naturally evolved minimal CTDs.

We propose that the high evolutionary conservation of the *T*. *brucei* CTDs might result from functions that are beyond VSG coat formation. Interestingly, we found that the type of CTD can influence the population doubling times of trypanosomes. The CTD-mediated modulation of parasite growth may be functional during antigenic variation. Antigenic switching to a “fast” VSG could be a clonal advantage in mixed populations, as “slower” clones would be outgrown. This could explain why distinct VSGs are temporally present in populations during an infection. On the other hand, slowdown of population growth could be advantageous in secluded environments such as the interstitial space, where high cell density would lead to quorum sensing-mediated cell cycle arrest and differentiation to the stumpy stage.

The question of how CTD can influence the population doubling time of the parasites can only be addressed by speculation. As the rate of VSG synthesis controls the cell division cycle, the CTD might modulate VSG translation. Alternatively, the rate of VSG recycling could be modulated by the type of CTD. Slower VSG recycling would lead to decreased endocytic uptake of nutrients, which in turn could slow down cell proliferation.

The reaction of the parasites to overexpression of VSGs that are slightly smaller than a minimal VSG may be reflective of a so far unknown facet of VSG control. Within 6 h of induction of expression, the mutant protein is displayed on the trypanosome cell surface and the mRNA is expressed at wild-type levels. At the same time cell proliferation ceases and the number of G2-arrested cells increases. The G2-arrest is reminiscent of the phenotype following ablation of VSG mRNA by RNAi, namely rapid pre-cytokinesis arrest and cell death. This has been interpreted as the VSG protein or *VSG* mRNA being monitored during cell cycle progression (11). Subsequent experiments using morpholinos showed that lack of protein rather than mRNA was responsible for the phenotype (12). In our experiments, expression of sub-minimally sized VSG mutants also resulted in rapid pre-cytokinesis arrest; however, the mutant proteins were made, possessed a GPI-anchor and were incorporated into the plasma membrane. Thus, these subminimal CTD deletion mutants are, in principle, compatible with the VSG coat. Furthermore, the native VSG mRNA decreased in the first 6 h of overexpression of the VSG mutant, which is likewise observed after overexpression of a wild-type VSG. Thus, on the mRNA level, there was no obvious phenotypic difference. The production of VSG-coated membrane continued. The new membrane, however, could not be accommodated on the cell surface, as cell growth was stalled. A possible enlargement of the ER was not observed. Instead, the only cell surface compartment that is not tethered to the cytoskeleton, the flagellar pocket, was enlarged. Swelling of the flagellar pocket has been observed in various unrelated studies and may have different causes (43, 44, 45). In the case of overexpression of sub-minimally sized VSGs, the reason for flagellar pocket enlargement appears to be the surplus of total VSG protein. Our data agrees with a scenario in which VSG translation triggers plasma membrane synthesis. The new coat is routed towards the flagellar pocket, where it fuses with the existing cell surface. Native and mutant VSGs form a mixed cell surface coat, which means that protein diffusion in and out of the pocket is occurring. At early time points, no disruption of the endomembrane system was observed by electron microscopy, and clathrin-mediated endocytosis seemed not to be impaired. At later time points, the swelling of the flagellar pocket led to a loss of intracellular order, possibly affecting many cellular processes. When we quantified the membrane area of the enlarging pocket, we found that the added area was equivalent to one complete trypanosome cell surface.

This leaves us with the central question—what triggers the phenotype? The sub-minimally sized VSG is biosynthesized, GPI-anchored and trafficked like a wild-type VSG. It is not recognized as non-functional until it has become part of the VSG surface coat. The fact that VSGs with completely randomized minimal CTDs were expressed and supported cell growth shows that the amino acid sequence does not matter as long as a certain domain size and flexibility is assured. Our results are compatible with a hypothetical scenario in which VSG quality control occurs during VSG recycling, when the VSG-coat density is transiently diluted 10-fold. This putative control step could be activated very rapidly after incorporation of the sub-minimally sized VSG into the cell surface, as membrane recycling in trypanosomes is exceptionally fast (9). We hypothesize that mechanosensors within the inner endosomal membrane could be triggered by VSGs that contain sub-minimally sized C-terminal domains. The detection of VSGs that are regarded too small leads to a very stringent cellular reaction, namely, cell cycle arrest without halt of VSG synthesis. Although this possibility is clearly pure speculation, it is consistent with the data.

In conclusion, we have found that the structurally conserved C-terminal domains of *T*. *brucei* VSGs are not essential *in vitro*. We further showed that a minimal CTD simply needs to be sufficiently large and flexible, probably to lift the N-terminal domain to a certain height over the plasma membrane to allow for diffusion-limited function. If these minimal length requirements are not met, the cell still produces the sub-minimal VSG, which however, is probably “detected” as defective shortly after incorporation into the surface coat, resulting in terminal cell cycle arrest. We surmise that the selective pressure to keep the conserved CTDs of *T*. *brucei* VSGs comes from functions not related to VSG structure, such as control of population doubling time.

## Experimental procedures

### Generation of VSG mutants

VSG mutants were either generated by PCR mutagenesis using the respective wild-type DNA as a template or synthesized (GeneCust, France). Mutagenesis by PCR was performed following the method published by Heckman and Pease (46). In brief, two separate PCR products were generated using Phusion DNA polymerase (Thermo Fisher Scientific) with a wild-type VSG-carrying pBluescript SK (+) (pBSK) plasmid serving as the template and amplification primer pairs T3/mutagenesis primer (reverse) or T7/mutagenesis primer (forward). These two products then served as templates and primers in a second PCR to generate the full-length VSG mutant which was amplified with T3 and T7 primers. See Table S1 for a full list of VSG mutants generated with specification of method employed (including mutagenesis primer sequences when PCR mutagenesis was used) and the full DNA and protein sequences. PCR products were cloned into the pJET1.2 cloning vector (Thermo Fisher Scientific), and successful mutant generation was confirmed by DNA sequencing (GATC/Eurofins). VSG mutants generated were then subcloned into a pBSK vector and mobilized from here with HindIII and SmaI and ligated into a pLew82v4 vector (HindIII/XhoI(blunt)) (Addgene plasmid #24009) for overexpression studies. The pKD vector (47) which integrates upstream of the endogenous MITat1.2 VSG in the 221-expression site was used for constitutive expression from the active expression site. To remove the endogenous MITat1.2 VSG from the active expression site, the knockout vector 221KOpuro was used which replaces the VSG with a puromycin resistance cassette.

### *T*. *brucei* cultivation and transfection

Bloodstream form *T*. *brucei*, cell line 13.90 (Lister 427) expressing VSG MITat1.2 served as the parental cell line in this study (48). Cells were cultivated at 37 °C and 5% CO_2_ in HMI-9 medium supplemented with 10% (v/v) heat inactivated fetal calf serum. Maintenance of the T7-polymerase and tetracycline repressor was achieved by selection with 5 μg/ml hygromycin and 2.5 μg/ml G418. For transfection, 10 μg of linearized plasmid DNA (pLEW82v4 (Addgene plasmid #24009): NotI, pKD (47): BglII, 221KOpuro: XhoI) was introduced into 3 x 10^7^ cells resuspended in 100 μl Amaxa Basic Parasite Nucleofector Kit 1 solution (Lonza) by electroporation using program X-001 of an AMAXA Nucleofector II (Lonza). Limiting dilution immediately following transfection ensured the generation of independent clones. Selection for successfully transfected cells (1 μg/ml phleomycin for pLew82v4 constructs, 30 μg/ml G418 for pKD constructs and 3 μg/ml puromycin for the 221KO construct) was added approximately 6 h post transfection. Induction of expression was achieved by the addition of 1 μg/ml tetracycline.

### *T*. *brucei* growth analysis

To assay growth of the trypanosome cell lines generated, cells were counted manually with a hemocytometer. For each cell line 3 independent clones were analyzed both without and with induction of expression of the mutant VSG. Cells were counted pre-induction with tetracycline (0 h) and at timepoints 6 h, 12 h, 24 h, and 48 h post induction.

### *T*. *brucei* volume measurements

The cell volume of cells expressing a particular VSG of interest was analyzed pre-induction (0 h) and 24 h and 48 h post-induction of expression with tetracycline with a Coulter counter Multisizer 4e equipped with a 50 μm aperture tube (Beckman Coulter GmbH, Germany). To avoid clogging of the orifice, cells were cultured in 0.2 μm filtered medium. At each timepoint, a total of 30,000 cells were analyzed in a size range of 3.0 to 30 μm equivalent spherical diameter (ESD).

### Immunofluorescence analysis

For immunofluorescence analyses 4 x 10^6^ cells each were harvested by centrifugation at 1400×*g* and room temperature (RT) for 10 min, washed once in HMI-9 without serum and resuspended in 1 ml of the latter. Cells were fixed in 4% formaldehyde solution (final concentration; prepared freshly from paraformaldehyde). Fixed cells were left to settle on poly-L-lysine coated slides for 1 h and washed with PBS, incubated for 10 min with 100 mM Tris-HCl and washed again with PBS. Samples were then blocked for 1 h with 1% BSA followed by incubation for 1 h with the primary antibody rabbit anti-MITat1.6 (1:1000; generated by BJ-Diagnostik, based on provided purified MITat1.6 protein). After three 5-min washes with PBS the cells were incubated for 1 h with a secondary Alexa488-conjugated anti-rabbit antibody (1:500; Thermo Fisher Scientific) and subsequently washed four times for 5 min with PBS. For staining of nuclei and kinetoplasts, the samples were incubated for 5 min with DAPI (500 ng/μl solution) and washed once in PBS prior to mounting with 80% glycerol. Specificity for VSG MITat1.6 was shown by including the parental MITat1.2 expressing cell line 13.90 as a negative control. Images were acquired with a DMI6000 B widefield microscope (Leica Microsystems) using an HCX PL APO CS objective (100x, NA = 1.4, Leica Microsystems) and Type F immersion oil (refractive index = 1.518, Leica Microsystems). LAS-X software (Leica Microsystems) was used to control the microscope. An EL6000 light source containing a mercury short-arc reflector lamp (HXP-R120 W/45C VIS, OSRAM) was used for illumination. Excitation light was selected by using GFP (470/40 nm) and A4 (360/40 nm) bandpass filter cubes (Leica Microsystems). Emitted light was collected at ranges of 525/50 nm (GFP) and 470/40 nm (DAPI). Identical settings for exposure times and camera gains were used for the different samples. Data were acquired with a DFC365 FX monochrome CCD camera (Leica Microsystems; 6.45 μm pixel size) and 150 z-slices with step-size = 100 nm were acquired. Acquired data were deconvolved using Huygens Essential software (Scientific Volume Imaging BV). Images were prepared using FIJI (49) and are displayed as maximum intensity projections.

### Fluorescence labeling of the trypanosome cell surface

1 x 10^7^ cells were harvested by centrifugation for 10 min at 1400×*g* and 4 °C, washed with trypanosome dilution buffer (TDB; 5 mM KCl, 80 mM NaCl, 1 mM MgSO_4_, 20 mM glucose, 20 mM Na_2_HPO_4_, 2 mM NaH_2_PO_4_, pH 7.6), and following centrifugation at 2000×*g* at room temperature (RT) for 90 s, the cell pellet was resuspended in 100 μl TDB to a concentration of 1 x 10^8^ cells/ml. Atto 488-NHS-ester (ATTO-TEC GmbH; 10 mM in DMSO) was added to a final concentration of 100 μM and cells were incubated in the dark for 15 min on ice. Following this, cells were washed 3 times with TDB to remove unbound dye (2000×*g*, RT, 90 s) and fixed in a final concentration of 2% formaldehyde prepared freshly from paraformaldehyde.

For imaging, the fixed cells were then settled onto poly-L-lysine coated coverslips (prepared by cleaning the coverslips with 70% EtOH, coating with 0.01% poly-L-lysine for ≥20 min and drying) by centrifugation at 750×*g* and RT for 1 min. The coverslips were mounted onto glass slides with Vectashield containing DAPI (Vector Laboratories) and images were acquired using a DMI8 widefield microscope (Thunder Imager, Leica Microsystems) with an HCX PL APO CS objective (100x, NA = 1.4, Leica Microsystems) using Type F Immersion Oil (refractive index = 1.518, Leica Microsystems). The microscope was controlled by the LAS-X software (Leica Microsystems). An LED8 light source was used for sample illumination (Leica Microsystems). Excitation light was selected by using the filter set: Ex 436/28 nm; DC 459 nm; Em 519/25 nm (Atto488). Images were captured using a K5 sCMOS camera (6.5 μm pixel size, Leica Microsystems). The single plain images acquired were processed by instant computational clearing (option provided by the Thunder Imager) and figures were prepared with FIJI (49).

### Flagellar pocket modeling

Cells were labeled with Atto 488-NHS-ester as described earlier. Image stacks (150 images with 100 nm z-steps) for subsequent modeling of the cell surface and the flagellar pocket surface were acquired on an iMic wide field fluorescence microscope (FEI Munich (formerly TILL photonics) equipped with a CCD camera (Sensicam qe, 6.45 μm pixel size, PCO) using a 100x objective (NA 1.4) (Olympus).

3D surface modeling and surface measurements were performed in Imaris (Oxford Instruments) and Amira (Thermo Fisher Scientific). Deconvolved image stacks of membrane-stained trypanosomes were analyzed as 3D volume models in Imaris and surface models were produced with threshold settings allowing either the modeling of the surface membrane or the modeling of flagellar pockets. For the latter models, a semi-automatic watershed algorithm was used in Imaris, separating local maxima of the original membranous fluorescence signal and the visualized surface domains of the pockets were selected manually. All surface models were exported separately as VRML files. These files were imported with Amira to complete surface modeling using the surfacewrap function. Based on these models the surface area of the cells and flagellar pockets were measured. Figures were generated by the combination of the cell surface and flagellar pocket models.

### Cell cycle and FP analysis

For analysis of the cell cycle, cells were chemically fixed in 2% formaldehyde and the nuclei and kinetoplasts were stained with DAPI (see above). At least 300 cells were analyzed per timepoint and separated according to the amount of kinetoplasts (K) and nuclei (N) present in the cell as 1K1N, 1Kd1N (dividing kinetoplast), 2K1N, 2K2N, and for aberrant cells as 1K2N, 1Kd2N, or “other.”

For a combined analysis of the cell cycle and the flagellar pocket size, cells were surface labelled with NHS-Atto488 (as above), chemically fixed in 2% formaldehyde and the nuclei and kinetoplasts stained with DAPI (see above). Cells were picked from array images (100 images with 100 nm z-steps) and the nucleus and kinetoplast configuration determined. Flagellar pocket sizes were determined with FIJI from z-projections by measuring the two-dimensional surface areas of the individual flagellar pockets and calculating the equivalent spherical diameters which are given as flagellar pocket diameters.

### Live cell imaging of trypanosomes

For live cell imaging, cells were concentrated by centrifuging 1 ml of culture for 90 s at 2000×*g* and RT. The sample was applied to a slide and covered by a coverslip. Cells were imaged with a DMI6000 B widefield microscope (Leica Microsystems) using an HCX PL APO CS objective (100x, NA = 1.4, Leica Microsystems) as described above, though the CMOS camera pco.edge (PCO, Kelheim) was used for data acquisition. For displaying, single frames were extracted from the short videos acquired.

### Electron microscopy

Samples for analysis *via* electron microscopy were either chemically fixed or high pressure frozen and embedded in Epon. Cells for chemical fixing were harvested by centrifugation at 1400×*g* and 4 °C for 10 min, washed once in TDB, resuspend in TDB and fixed in 2% formaldehyde and 0.05% glutaraldehyde. Cells were then washed in 50 mM cacodylate buffer before contrasting with 2% OsO_4_ in cacodylate buffer for 1 h. After washing with water, the cells were further contrasted overnight with 0.5% uranyl acetate and washed with water. Cells were then dehydrated with ethanol and embedded in Epon. For high-pressure freezing, at least 3 x 10^7^ cells were harvested by centrifugation for 3 min at 750×*g* and RT. Fetal calf serum was added to the cells as a cryoprotective prior to high-pressure freezing, with samples exposed to > 2100 bars of pressure and cooling rates of > 20,000 K s^-1^ (EM HPM100, Leica Microsystems). Freeze substitution was performed in a Leica EM AFS2 (Leica Microsystems) based on protocols available in the literature (50, 51), and cells were subsequently embedded in Epon. Images were acquired from 65 nm slices on either an EM900 or EM10 transmission microscope (Carl Zeiss AG).

### RNA extraction and quantification of VSG mRNA

Total RNA was isolated from 1 x 10^8^ trypanosomes harvested by centrifugation for 10 min at 1400×*g* and 4 °C using a Qiagen RNeasy Mini Kit (Qiagen) according to the manufacturer’s instructions. For RNA quantification, 3 μg of total RNA was denatured with deionized glyoxal for 40 min at 50 °C and blotted onto an Amersham Hybond-N membrane (Cytiva) using a Minifold Dotblotter (Schleicher & Schuell). Following UV crosslinking of the RNA to the membrane (1200 x 100 μJ/cm^2^) the membrane was deglyoxylated by incubating for 1 h at 80 °C. After prehybridizing the membrane for 1 h at 42 °C in hybridization solution (5x SSC (750 mM NaCl, 75 mM tri-sodium citrate, pH 7.0), 5x Denhardt’s solution (0.1% (w/v) BSA, 0.1% (w/v) polyvinylpyrrolidone, 0.1% (w/v) Ficoll), 0.1% (w/v) SDS, 0.01% (w/v) heparin, 3.8 mM tetrasodium pyrophosphate) fluorescently labelled oligonucleotide probes (VSG121-probe: IRDye 682-GGCTGCGGTTACGTAGGTGTCGATGTCGAGATTAAG or VSG221(ORF)-probe: IRDye 682-CAGCGTAAACAACGCACCCTTCGGTTGGTCGTCTAG or VSG221(5′ UTR)-probe: IRDye 682-TTCGTGTCGCGTAGGAATAACTACAA together with a Tubulin-probe: IRDye 782-ATCAAAGTACACATTGATGCGCTCCAGCTGCAGGTC; all synthesized by Eurofins) were added and left to incubate overnight at 42 °C and in the dark to allow detection and quantification of VSG mRNA together with tubulin mRNA which served as a loading control. Blots were washed twice with 0.1% SDS and 0.1x SSC before drying. Blots were visualized using a LI-COR Odyssey Imaging System (LI-COR Biosciences) and analyzed using Image Studio Lite software (LI-COR Biosciences).

### Visualization and quantification of VSG protein in whole cell lysates

Whole cell lysates were generated by harvesting a defined number of cells by centrifugation for 10 min at 1400×*g* and 4 °C, washing them once with ice-cold trypanosome dilution buffer (TDB; 5 mM KCl, 80 mM MgSO_4_, 20 mM Na_2_HPO_4_, 2 mM NaH_2_PO_4_, 20 mM glucose, pH 7.6) and resuspending in protein loading buffer (2% (w/v) SDS, 10% (v/v) glycerol, 60 mM Tris-HCl, pH 6.8 with 1% (v/v) β-mercaptoethanol) to the desired cell density followed by boiling for 5 min at 100 °C. For visualization, proteins from 5 x 10^5^ cell equivalents were separated together with a size standard (PageRuler Prestained Protein Ladder, 10–180 kDa, Thermo Fisher Scientific) on a 12.5% sodium dodecycl sulphate (SDS)-polyacrylamide gel with subsequent transfer to a nitrocellulose membrane (Cytiva). Alternatively, specifically when quantifying, the protein was applied directly to the membrane using a Minifold Dotblotter (Schleicher & Schuell). Membranes were blocked overnight at 4 °C with 5% skimmed milk powder (milk) in PBS (137 mM NaCl, 2.68 mM KCl, 8.09 mM Na_2_HPO_4_, 1.76 mM KH_2_PO_4_, pH 7.4). Membranes were incubated with primary antibodies—polyclonal rabbit anti-MITat1.2 (1:5000; made by BJ-Diagnostik, based on provided purified MITat1.2 protein) or polyclonal rabbit anti-VSG121 (1:2,000, MITat1.6 detection; kindly provided by Mark Carrington) and for a loading control monoclonal mouse anti-PFR (L13D6 (52), 1:20)—in 1% milk and 0.1% Tween-20 in PBS for 90 min at RT. Removal of unbound antibodies was achieved by washing the membranes 4 x for 5 min each in 0.2% Tween-20 in PBS. Fluorophore-coupled secondary antibodies—IRDye800CW-conjugated goat-anti-rabbit and IRDye680LT-conjugated goat-anti-mouse (both 1:10,000, LI-COR Biosciences)—were added in 1% milk and 0.1% Tween-20 in PBS and incubated for 90 min at RT in the dark. Removal of unbound antibodies followed with four 5 min washes in 0.2% Tween-20 in PBS and one 5 min wash in PBS. Proteins were visualized using a LI-COR Odyssey Imaging System (LI-COR Biosciences) and analyzed using the Image Studio Lite software (LI-COR Biosciences).

### Small-scale Triton X-114 cell lysis and phase separation of protein

Triton X-114 cell lysis and phase separation of proteins essentially followed the protocols published in (33, 34). 1.5 x 10^7^ cells were harvested by centrifugation for 10 min at 1.400×*g* and 4 °C and washed once in 1 ml of ice-cold TDB (90 s, 2000×*g*, RT). The cell pellet was then resuspended in 450 μl of lysis buffer (2% Triton X-114 in PBS (prepared from pre-condensed Triton X114) supplemented with protease inhibitor cocktail (cOmplete, EDTA-free, #04693132001, Roche), and the cell suspension vortexed for 30 s and then incubated on ice for 30 min with occasional ∼5 s bouts of vortexing (3–4 times in total). The cell suspension was then centrifuged for 2 min at 16,100×*g* and 4 °C to remove cell debris. The supernatant was transferred to a fresh 0.5 ml reaction tube and incubated for 10 min at 37 °C in a water bath. Phase separation was achieved by centrifugation for 10 min at 30 °C and 21,100×*g*. The upper aqueous phase was carefully removed and placed into a fresh reaction tube.

Protein samples were prepared from 135 μl each of the cell lysate and the supernatant of the cell lysate, and from both the resulting aqueous and Triton X-114 phases. For this, the Triton phase sample was adjusted to a volume of 100 μl by the addition of water, and the proteins were extracted as follows with chloroform/methanol. First, 400 μl of methanol was added and the mixture vortexed. Then 100 μl of chloroform was added and the mixture again vortexed. Following this, 300 μl (if the initial sample had a volume over 100 μl, the excess was subtracted at this point) of ice-cold water was added and the sample again vortexed. Centrifugation for 1 min at 14,000×*g* and 4 °C yielded three phases, with the protein precipitate at the interface of the upper aqueous and lower chloroform phase. The aqueous phase was carefully removed, and a further 400 μl of methanol was added, and the sample was vortexed. Following this, the proteins were precipitated by centrifugation for 5 min at 20,000×*g* and 4 °C. The supernatant was carefully removed, the precipitate dried, protein sample buffer containing β-mercaptoethanol added to give a final concentration of 1 x 10^5^ cell equivalents/ml, and the sample boiled for 5 min at 100 °C. Samples were analyzed by SDS-PAGE on a 10% acrylamide gel. Proteins were visualized by staining with Coomassie Brilliant Blue R-250. Images were acquired on an iBright Imager (Thermo Fisher Scientific).

## Data availability

All constructs and cell lines are available from the authors.

## Supporting information

This article contains supporting information.

## Conflict of interest

The authors declare that they have no conflicts of interest with the contents of this article.
