# Supplementary material for: Structure–function analysis defines the minimal functional C-terminal domain of the variant surface glycoprotein of Trypanosomabrucei
Source: J Biol Chem. 2025 May 22;301(7):110260. doi: 10.1016/j.jbc.2025.110260 (PMC12226131; doi:10.1016/j.jbc.2025.110260)
Supplement: Supporting Information [file mmc1.pdf]

Structure-function analysis defines the minimal functional C-terminal domain of the variant surface glycoprotein of *Trypanosoma brucei*

Nicola G. Jones*, Markus Engstler*

Department of Cell and Developmental Biology, University of Würzburg, Würzburg, Germany

*corresponding authors: nicola.jones@uni-wuerzburg.de; markus.engstler@uni-wuerzburg.de

Fig. S1: High level ectopic expression of both MITat1.6 and MITat1.2 support growth of *Trypanosoma brucei*. *A*, comparison of the type 1 C-terminal domains of VSGs I1.24 and M1.6 which share the same overall structure and a relatively high sequence similarity. Barrels above the sequences indicate determined (black) and inferred (grey) α- or 3_{10}-helices, arrows stand for β-sheets. NTD, N-terminal domain, S1 and S2, first and second structured domain within the C-terminal domain, respectively. N-glycosylation sites are highlighted in green and conserved cysteines in yellow. *B*, ectopic expression of wild-type VSGs M1.6 (W1) and M1.2 (W2) using a tetracycline-inducible expression system with a strong T7 promoter over a time of 168 h. Growth of uninduced cells is shown in black, with growth of induced cells shown in green for M1.6 (W1) and in blue for M1.2 (W2). Growth was observed for three independent clones each and is displayed as the average with error bars showing the standard deviation. Guidelines at 10^6 and 10^{10} cells are shown to highlight the different growth rates of cells induced to overexpress W1 or W2. *C*, RNA dot blots (left) showing that ectopic *VSG M1.2* expression led to downregulation of the endogenous *VSG M1.2* mRNA. Total *M1.2* mRNA and endogenous *M1.2* mRNA were detected using fluorescently labeled probes and normalized to *β-tubulin* mRNA. Quantification in the graph is given relative to the parental *M1.2* expressing 13.90 cell line. Ectopic *VSG M1.2* amounts were determined indirectly by subtraction of the endogenous *VSG M1.2 mRNA* amounts from the total *VSG M1.2* mRNA amounts.

Fig. S2: The C-terminal domain of *T. brucei* VSGs requires a minimal size to support cell growth whilst the composition of the linker L1 is flexible. Growth curves over a time period of 168 h showing the effect of overexpression of mutants D17, D18 and D19. Growth of uninduced M1.2 wt expressing cells is shown in black, with growth of cells induced to express the respective M1.2 mutant shown in blue. Growth was observed for three independent clones each and is displayed as the average with error bars showing the standard deviation. Guidelines at 10^6 and 10^{10} cells are shown to highlight the different growth rates of cells induced to overexpress D17 and D19 (similar to W2) and D18 (similar to W1).
