# Supplementary figures and images for: Structure–function analysis defines the minimal functional C-terminal domain of the variant surface glycoprotein of Trypanosomabrucei
Source: J Biol Chem. 2025 May 22;301(7):110260. doi: 10.1016/j.jbc.2025.110260 (PMC12226131; doi:10.1016/j.jbc.2025.110260)

**A**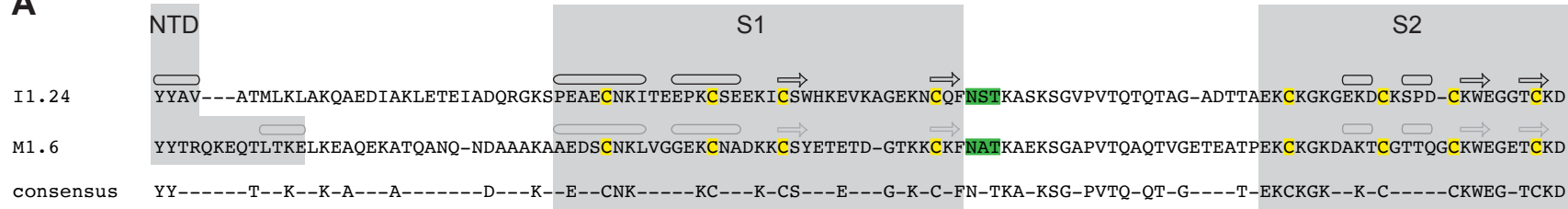**B**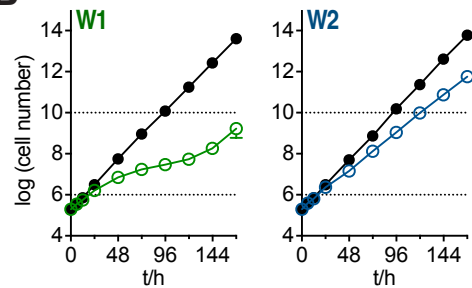**C**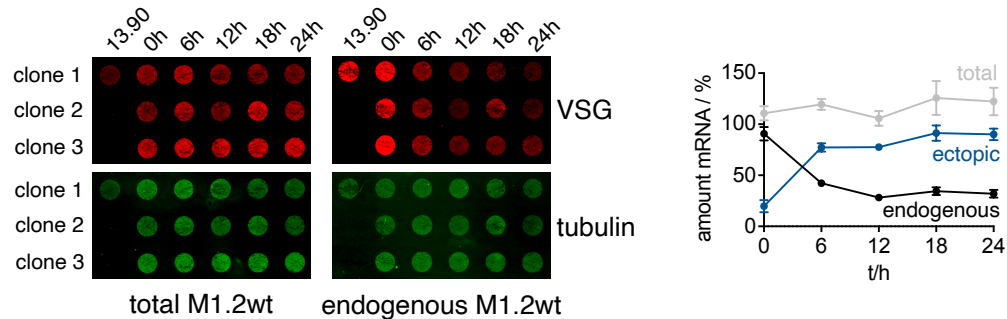**Figure S1**

Supplement: Figure S1 [file mmc3.pdf]

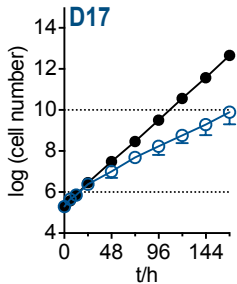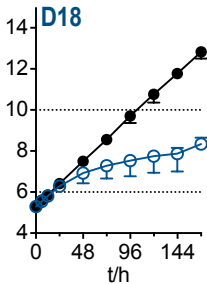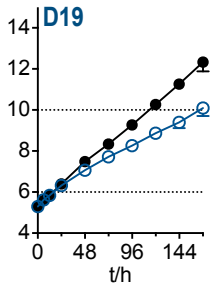

**Figure S2**

Supplement: Figure S2 [file mmc4.pdf]
